# A review on the promising antibacterial agents in bone cement–From past to current insights

**DOI:** 10.1186/s13018-024-05143-7

**Published:** 2024-10-20

**Authors:** Hao Lin, Zhe Gao, Tao Shan, Ayakuzi Asilebieke, Rui Guo, Yu-chen Kan, Chun Li, Yang Xu, Jian-jun Chu

**Affiliations:** 1 Department of Orthopedics, The Second People’s Hospital of Hefei, Hefei Hospital Affiliated to Anhui Medical University, Hefei, 230011 Anhui China; 2 Department of Orthopedics, Hefei BOE Hospital, Teaching Hospital of Shanghai University Medical College, Hefei, 230013 Anhui China; 3 Department of Orthopedics, The First People’s Hospital of Hefei, The Third Affiliated Hospital of Anhui Medical University, Hefei, 230000 Anhui China; 4 Department of Pharmaceutical Science and Engineering, School of Food and Biological Engineering, Hefei University of Technology, Hefei, 230009 Anhui China

**Keywords:** Bone cements, Antibacterial agents, Antibiotics, Antimicrobial peptides, Tissue adhesives

## Abstract

Antibacterial bone cements (ABCs), such as antibiotic-loaded bone cements (ALBCs), have been widely utilized in clinical treatments. Currently, bone cements loaded with vancomycin, gentamicin, tobramycin, or clindamycin are approved by the US Food and Drug Administration. However, traditional ALBCs exhibit drawbacks like burst release and bacterial resistance. Therefore, there is a demand for the development of antibacterial bone cements containing novel agents to address these defects. In this review, we provide an overview and prospect of the new antibacterial agents that can be used or have the potential to be applied in bone cement, including metallic antibacterial agents, pH-switchable antibacterial agents, cationic polymers,* N*-halamines, non-leaching acrylic monomers, antimicrobial peptides and enzymes. Additionally, we have conducted a preliminary assessment of the feasibility of bone cement containing *N*-halamine, which has demonstrated good antibacterial activities. The conclusion of this review is that the research and utilization of bone cement containing novel antibacterial agents contribute to addressing the limitations of ALBCs. Therefore, it is necessary to continue expanding the research and use of bone cement incorporating novel antibacterial agents. This review offers a novel perspectives for designing ABCs and treating bone infections.

**Graphical abstract:**

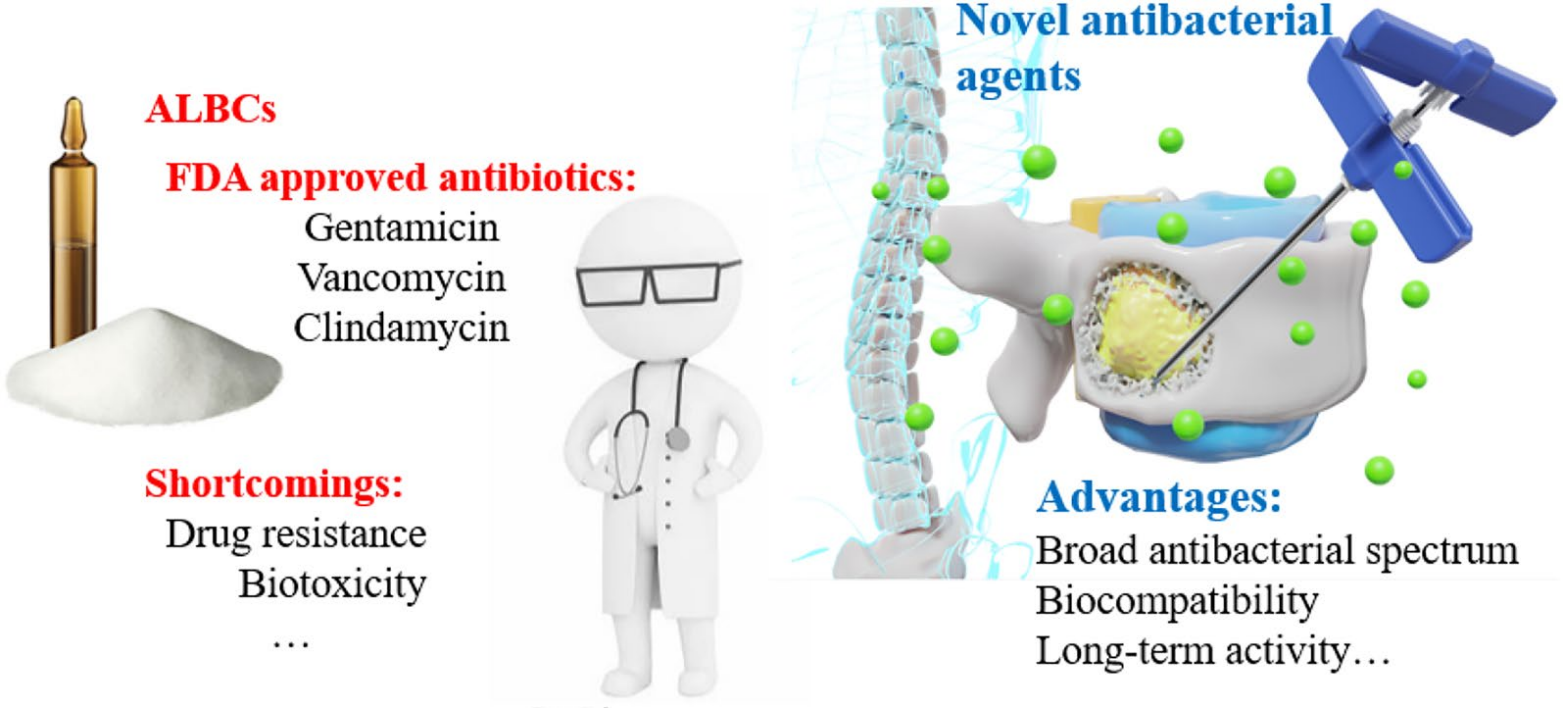

## Introduction

With factors such as global aging, military and civilian trauma, orthopedic surgeries are increasing [1]. In 2019, the number of total knee and hip replacement surgeries in China reached more than 950,000 cases [2]. In the United States, more than 1 million joint replacement surgeries are performed annually [3], and it is predicted that by 2030, the demand for total hip and total knee arthroplasties in the United States will increase to over 4 million cases [4].

Despite preoperative and prophylactic antibiotic treatment, infections related to invasive orthopedic surgeries are common, exceeding 25% in complex open fractures or revision surgeries [5]. In addition, data show that 5% to 10% of joint replacements require revision within 7 years, with prosthetic joint infection (PJI) being one of the most common reasons for revision [6]. PJI is a severe complication of prosthetic replacement with a recurrence rate of 16% and a mortality rate of 2.5% [7, 8]. In the treatment of bone infections, achieving effective concentrations of antibiotics at the site of infection through intravenous or oral routes can be challenging due to the poor blood supply to the infected area. This has led to the implantation of antibacterial bone cements (ABCs) as a significant treatment option for orthopedic infections [9]. Among them, antibiotic-loaded bone cements (ALBCs) are the most widely used and studied type of antibacterial bone cement as it is the gold standard for use in orthopaedic procedures [10, 11]. It is widely believed that, in comparison to systemic antibiotic therapy, the local release of antibiotics from ALBCs can achieve significantly higher local concentrations of antibiotics directly at the site of infection [12].

Bone cement is an injectable orthopedic biomaterial with self-setting properties. According to the type of material, bone cement can be divided into two categories: 1) organic bone cement, mainly polymethylmethacrylate (PMMA) bone cement; 2) inorganic bone cement, represented by calcium phosphate cement (CPC), calcium sulfate cement (CSC), etc. PMMA bone cement has the advantages of high mechanical strength and good injectability, while CPC and CSC have poor mechanical strength but better biocompatibility and biodegradability[13, 14]. PMMA based antibiotic-eluting bone cement has gradually become the gold standard for ALBCs due to its excellent mechanical properties and injectability[15, 16]. Currently, bone cements loaded with vancomycin, gentamicin, tobramycin, or clindamycin are approved by the US Food and Drug Administration [17]. Heraeus Medical's COPAL® G + V contains gentamicin and vancomycin. Each 40 g of bone cement powder contains 0.5 g of gentamicin and 2 g of vancomycin. According to the product description, COPAL® G + V is specifically designed for the filling, stabilization, and permanent fixation of revised joint endoprostheses in surgically prepared bone cavities that have been previously infected with vancomycin-sensitive pathogens. It is indicated in scenarios where the use of gentamicin-only bone cement is deemed insufficient or not preferable during both single-stage and two-stage replacement procedures. Heraeus Medical's COPAL® G + C is a bone cement containing gentamicin and clindamycin. Each 40 g of bone cement powder contains 1 g of gentamicin and 1 g of clindamycin. COPAL® G + C is utilized for securely anchoring all compatible joint prostheses during primary arthroplasty procedures, offering enhanced protection against infection, as well as in revision surgeries necessitated by the aseptic loosening of the prosthesis or infection caused by gentamicin- and/or clindamycin-sensitive microorganisms.

While ALBCs have many appealing features, they also have their own drawbacks. One such drawback is that the most common bacteria causing orthopedic infections, *Staphylococcus aureus* (*S. aureus*) and *Staphylococcus epidermidis* (*S. epidermidis*), have gradually developed antibiotic resistance, particularly to gentamicin, which makes it difficult for the clinically common gentamicin bone cement to completely kill them [18, 19]. Although inorganic antibiotic-loaded bone cements can indeed exhibit prominent biological activity and osteogenic potential, it is also true that some antibiotics can inhibit osteoblast activity. Studies have shown that gentamicin, vancomycin, and other antibiotics have inhibitory effects on osteoblasts [20, 21]. Furthermore, the burst release exacerbates biological toxicity. And the subsequently long-term low-dose antibiotic release following the burst release can increase the risk of bacterial resistance. The addition of antibiotics can also lead to problems such as deterioration of mechanical strength and prolongation of setting time [22, 23]. Currently, to address the above mentioned problems, there have been many studies on novel antibacterial agents contained ABCs. For instance, ABCs containing silver can effectively kill drug-resistant bacteria while maintaining good cell compatibility, and ABCs containing quaternary ammonium polymer do not suffer from the issue of burst release [24, 25]. These studies on ABCs have greatly pushed forward the new horizon of orthopedic infection treatment.

**Fig. 1:**
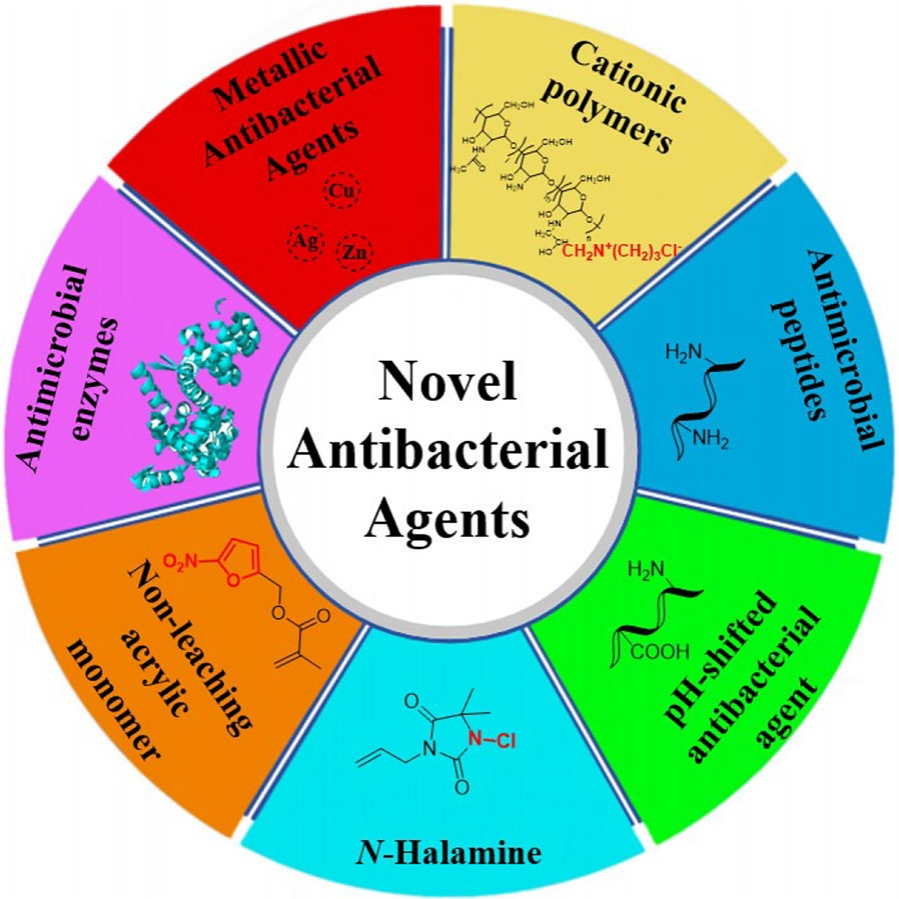
Antibacterial agents discussed in this review

The observations made above underscore the urgent need for the development of ABCs incorporating novel antibacterial agents. In this review, we aim to present an overview and prospect of bone cement loaded with antibacterial agents, encompassing metal ions, pH-switchable antibacterial agents, positively charged polymers, *N*-halamines, non-leaching antibacterial agents, antimicrobial peptides, and antimicrobial enzymes (Fig. 1). Our objective is to provide a survey of novel antibacterial agents that have been utilized in bone cement or have the potential to be incorporated into bone cement research, highlighting the ongoing issues in this area. This is the review of non-antibiotic added bone cement with the most types of new antibacterial agents. And this review is meant to highlight key concepts, and not meant to be comprehensive. Therefore, not all publications in the field are included. The authors encourage communications from colleagues to bring important omissions to the authors’ attention for inclusion in future updates.

### Metallic antibacterial agents

**Fig. 2:**
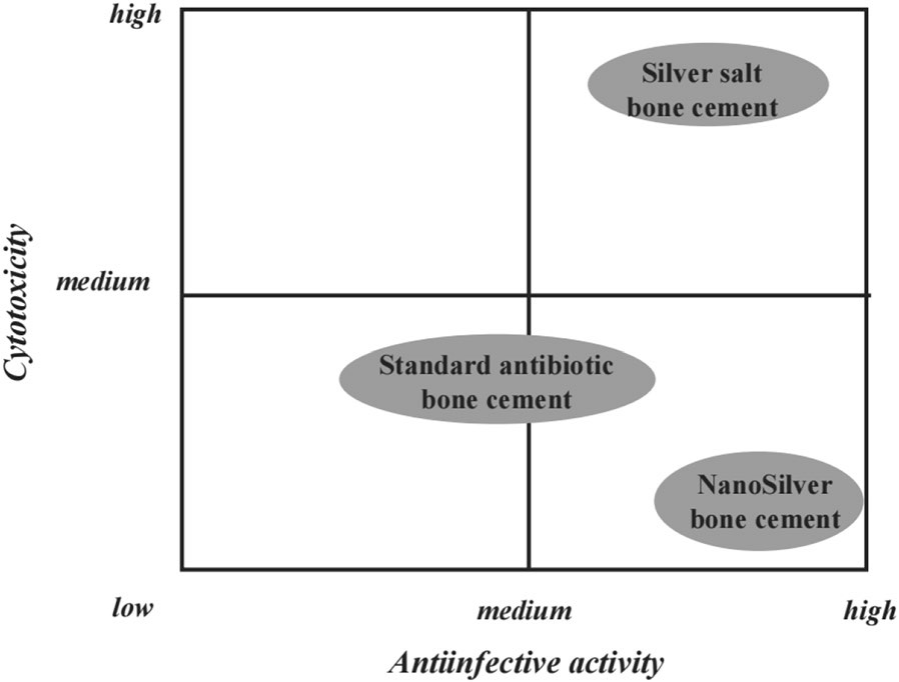
Overview of cytotoxictiy and antibacterial activity. Reprinted from Alt et al., Biomateri-als. 2004. 25: 4383–4391. Copyright 2003 Elsevier Ltd. Licensed under Creative Commons Attribu-tion 3.0 Unported

The widespread antibacterial activities of silver are well-known, and it has been utilized in various medical fields for years [26, 27]. Research on the antibacterial activities of silver, particularly silver salts, has persisted for decades [28]. For instance, Dueland et al. found that PMMA bone cement loaded with silver sulfate exhibited good in vivo antibacterial activity, enhancing survival rates in experimental animals [29]. However, numerous studies have also demonstrated that although silver salts possess antibacterial properties, they exhibit strong cytotoxicity when used internally. Consequently, recent research has focused on the development of nano-silver containing antibacterial bone cement with reduced biotoxicity [30]. Alt et al. discovered that acrylic bone cement loaded with nano-silver exhibited excellent antibacterial activity. Researchers compared the antibacterial activity and cytotoxicity of silver salt bone cement, gentamicin-loaded bone cement, and nano-silver bone cement (Fig. 2). This nano-silver cement showed good antibacterial activity against methicillin-resistant *S. epidermidis* (MRSE) and methicillin-resistant *S. aureus* (MRSA), while traditional gentamicin bone cement was ineffective against these resistant bacteria. Remarkably, this new nano-silver bone cement exhibited no cytotoxicity, marking a significant distinction from previously reported high cytotoxicity associated with silver salt bone cement. This may be due to the primarily non-leaching bactericidal effect of nano-silver, although the mechanism is currently unclear and may involve the generation of reactive oxidative species or cationic damage to the bacterial cell membrane. This differs from the bactericidal action of silver ions released by silver salts.

Beyond antibacterial properties, scientists are also interested in the bioactivity of silver-containing bone cement. Miola et al. prepared a silver containing bioactive glass powder (SBAG) and studied its antibacterial properties when added to bone cement [31]. Additionally, they examined the ability of this composite bone cement to form hydroxyapatite on its surface. They found that bone cement containing SBAG could release Ag ions, exhibiting good antibacterial activity, while also depositing hydroxyapatite on its surface, demonstrating certain bioactivity. Later, they doped silver ions with silica-based glass powder (SBA) to create a new bioactive glass, Ag-SBA2, which was then added to bone cement [32]. They discovered that this bone cement exhibited good antibacterial and bioactive properties, similar to those of SBAG containing bone cement. Remarkably, bone cement containing Ag-SBA2 also exhibited optimized mechanical strength.

In addition to silver, other metals such as copper, gold and zinc also possess antibacterial properties. Wekwejt et al. studied the cell compatibility of bone cement containing nano-silver and nano-copper [33]. They found that both silver and copper enhanced the antibacterial properties of the bone cement, but nano-silver containing bone cement exhibited excellent cell compatibility, while the cell compatibility of the copper containing group was poorer. For example, nano-silver containing bone cement had almost no hemolytic activity, while nano-copper containing bone cement exhibited higher hemolytic activity. Russo found that bone cement containing gold nanoparticles exhibited good antibacterial properties [34]. In addition, recent studies have shown that zinc and zinc oxide exhibit remarkable antibacterial activity [35]. Gholami et al. found that zinc oxide nanoparticles, particularly when coupled with quaternary ammonium compounds, significantly inhibited microbial growth in antimicrobial-resistant pathogens at low concentrations (MIC: 32 µg/mL) [36]. Recently, Eltohamy et al. created an antibacterial calcium silicate cement (CSC) containing zinc oxide that exhibited antibacterial activity against both Gram-positive (*S. aureus*) and Gram-negative *(Escherichia coli, E. coli*) bacteria[37].

Although PMMA bone cement is the most commonly used clinically, it has some limitations, such as its non-degradability. Therefore, recent research has focused on the development of silver containing degradable inorganic bone cement (such as CPC, brushite cement, etc.) [38–43]. Unlike the introduction of silver in PMMA bone cement, silver agents can be introduced through both the liquid and solid phases of inorganic bone cement. For instance, Jacquart et al. showed that the introduction of silver salts into the liquid or solid phase of calcium carbonatecalcium phosphate cement produced antibacterial and non-cytotoxic bone cement [39]. They found that using carboxymethylcellulose microspheres as a carrier for silver ions effectively avoided burst release of silver ions, thus minimizing their toxic effects [42]. This is an impressive recent advancement. Liu et al. comprehensively compared the antibacterial properties, cell compatibility, and injectability of silver ions and silver nanoparticles [43]. Their studies demonstrated that bone cement containing silver ions exhibited good injectability and cell compatibility. The higher cell compatibility of silver salts compared to nano-silver seems inconsistent with some previous understandings. We speculate that this may be due to the use of starch-modified CPC by Liu et al., which optimized the release profile of silver ions, avoiding burst release.

In conclusion, antibacterial bone cement containing metals, particularly silver (in the form of silver salts or nano-silver particles), exhibits good antibacterial properties (Table 1). In this active research field, onceinherent drawbacks, such as the biotoxicity of silver salts, are being overcome through innovative delivery methods. Recent studies have shown that metal ions can have biological activities beyond antibacterial activity, for example, zinc ions have cell proliferation and osteoinductive activities. Therefore, in addition to antibacterial properties, other biological activities of metal ions should also be studied.We believe that there will be more exciting metal containing antibacterial bone cements developed in the future (Table 2).

**Table 1:** Summary of the metallic antibacterial agents-loaded cements in the review

References	Metallic agents	Hosting cement type	Activity	Biocompatibility
[28, 29]	Silver salt	PMMA	*S. aureus, E. coli, Pseudomonas aeruginosa (P. aeruginosa)*	Cytotoxicity
[30]	Nano silver	PMMA	*Staphylococcus epidermidis (S. epidermidis),* MRSE, MRSA	Absence of in vitro cytotoxicity
[31, 32]	Silver-containing bioglass	PMMA	*S. aureus*	–
[33]	Nano silver	PMMA	*S. aureus*	Non-influential on erythrocyte hemolysis and blood platelet function
[33]	Nano copper	PMMA	*S. aureus*	Induces erythrocyte shape change and hemolysis, reduces platelet aggregation
[34]	Nano gold	PMMA	MRSA	–
[37]	Zinc oxide	CSC	*S. aureus, E. coli,*	Cell Proliferation Induction and Osteo-Inductivity Enhancement
[38, 39]	Silver salt	CPC	*S. aureus, S. epidermidis*	Non-influential on cell growth
[40]	Silver salt	CPC	*E. coli*	–
[42]	Silver-loaded microspheres	CPC	*S. aureus, S. epidermidis*	Non-cytotoxic
[43]	Silver salt	starch-modified CPC	*S. aureus*	Non-cytotoxic

**Table 2:** Summary of the AMPs-loaded cements in the review

References	AMPs	Hosting cement type	Activity	Biocompatibility
[57]	Dhvar-5	PMMA	MRSA	–
[59]	hLF1-11	CPC	MRSA	–
[65]	HDP mimicking peptide polymer	PMMA	MRSA	Low toxicity both in vitro and in vivo
[66, 67]	AMP with D-amino acids	PMMA	*S. aureus,* MRSA, *P. aeruginosa, S. epidermidis*	–

### Cationic polymers

Cationic polymers primarily refer to polymers containing quaternary ammonium compounds (QACs), which are extensively studied as antibacterial compounds [44–46]. The quaternary ammonium motifs carry positive charges and interact with the lipid membranes of Gram-negative and -positive bacteria. This electrostatic interaction with the negatively charged bacterial cell membranes leads to physical damage to the cells, conferring broad-spectrum antibacterial activity to these quaternary ammonium functionalized polymers. In recent years, QACs-containing antibacterial bone cement has made significant progress in the prevention and treatment of bone infections. Currently, the introduction of quaternary ammonium motifs can be achieved either through physical doping or covalent polymerization.

### Cationic polymers

Bone cement containing physically doped Cationic Polymers.

**Fig. 3:**
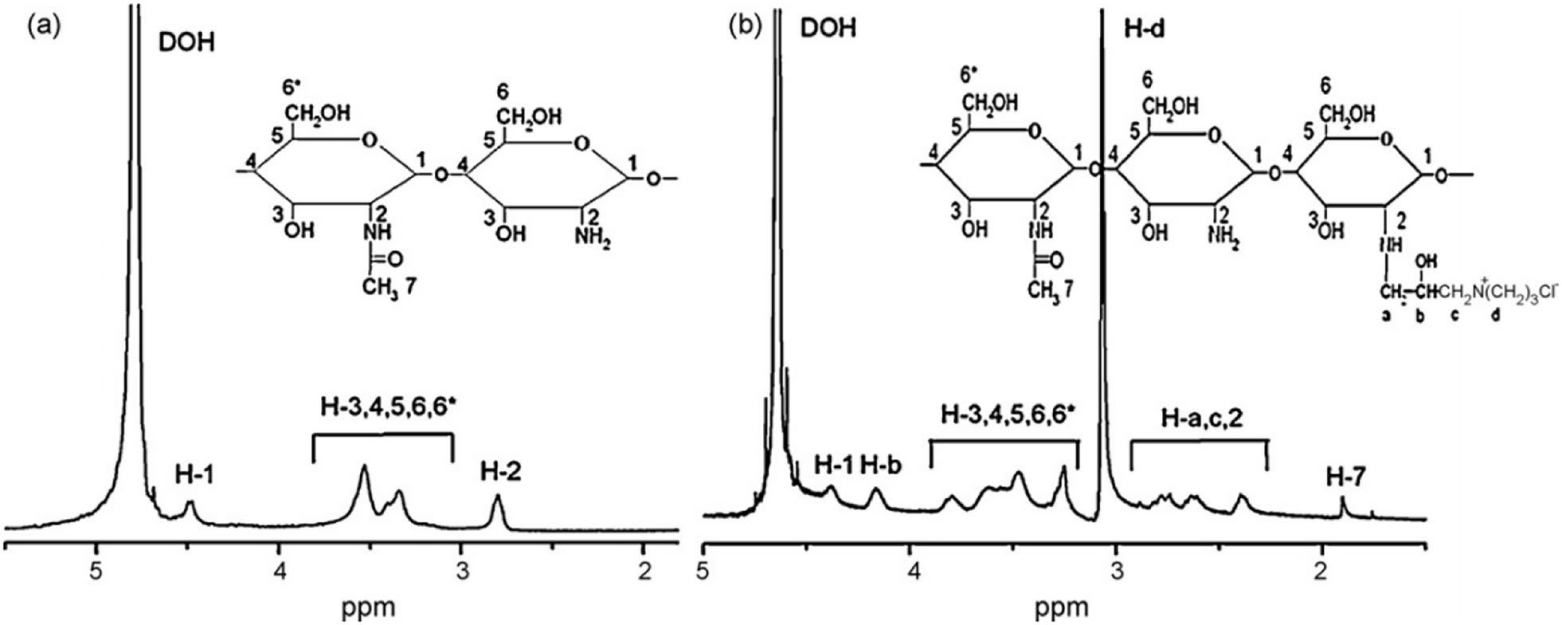
1H NMR spectrum of chitosan (**a**) and HACC 44% (**b**). Reprinted from Tang et al., Carbohyd Polym. 2010. 81: 275–283. Copyright 2010 Elsevier Ltd. Licensed under Creative Commons Attribution 3.0 Unported

**Fig. 4:**
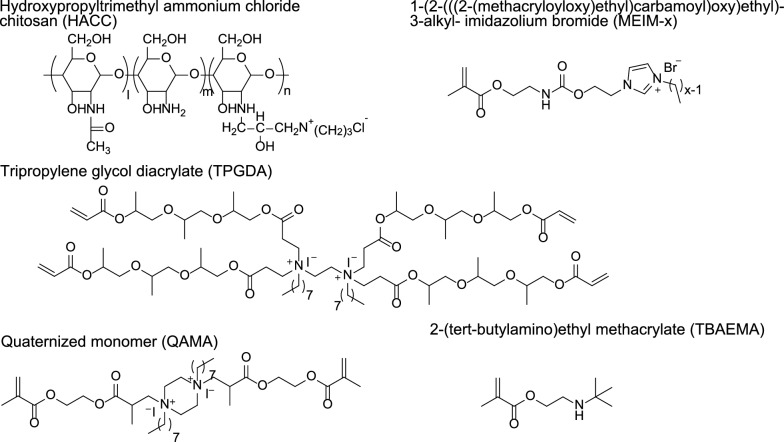
Cationic polymers or monomers containing QACs

Tang et al. designed hydroxypropyltrimethyl ammonium chloride chitosan (HACC) containing quaternary ammonium motifs (Figs. 3, 4) [47–49]. The addition of a certain amount of HACC to PMMA bone cement exhibits good antibacterial activity. Tang et al. found that HACC with a quaternary ammonium substitution degree of 26% exhibits good antibacterial activity and biocompatibility, while higher substitution degrees may cause biotoxicity. Furthermore, HACC bone cement exhibits superior ability to inhibit the formation of *Staphylococcus aureus* biofilm on the bone cement surface compared to gentamicin bone cement. Surprisingly, they also observed that this novel bone cement can downregulate the expression of virulence-related genes in antibiotic-resistant *Staphylococcus aureus*. To investigate the potential of this bone cement, Tang et al. conducted a dedicated biomechanical and biocompatibility study. The study showed that the optimal mass ratio of 26% substitution degree HACC added to PMMA bone cement is 20%, at which point the mechanical properties of the bone cement decrease significantly. After soaking for 4 weeks, the mechanical properties of the bone cement decrease slightly. Encouragingly, the mechanical strength of the HACC-loaded bone cement is close to that of human cancellous bone. Biocompatibility studies further revealed that HACC-loaded PMMA bone cement has good bone integration with bone tissue. Compared to chitosan or gentamicin-loaded bone cement, more new bone formation was observed around the HACC-loaded PMMA bone cement.

### Cationic polymers

Bone cement containing covalent polymeric cationic polymers.

In Tang et al.'s study, the quaternary ammonium motifs were first integrated into the glycan molecules, and HACC was considered as a biodegradable polymer of natural origin, and then non-covalently doped with PMMA bone cement. Many studies have also designed acrylic monomers containing quaternary ammonium motifs. These monomers are added to the liquid phase of the bone cement during use, forming bone cement containing QACs. Deb et al. developed an acrylic monomer, quaternized EGDMA-piperazine octyl ammonium iodide (QAMA), which has two polymerizable acrylic structures and can crosslink with PMMA bone cement [50]. It also contains two quaternary ammonium motifs, exhibiting antibacterial activity. When QAMA monomer is added as a component of the liquid phase to acrylic bone cement, the results show that bone cement containing 15% QAMA does not have a bacteriostatic zone, but there is no bacterial growth on the surface of the bone cement, demonstrating non-leaching antibacterial activity. The working mechanism of this antibacterial monomer is to eliminate bacteria through contact without releasing bioactive agents.

Singh et al. developed a 4-arm dendrimer quaternary ammonium salt acrylic acid comonomer with tripropylene glycol diacrylate (TPGDA), which indicates that it has four acrylic acid polymerizable structures (Fig. 4) [51]. Studies have shown that the powder of TPGMA cement has antibacterial activity. Although the surface area of the bone cement powder obtained by this grinding pretreatment is increased, which may be different from the morphology of the bone cement during use, it demonstrates its potential efficacy as an antibacterial agent in bone cement. He et al. developed acrylic acid copolymers containing imidazole quaternary ammonium salt, 1-(2-(((2-(methacryloyloxy)ethyl)carbamoyl)oxy)ethyl)-3-alkyl-imidazolium bromide (MEIM-x), which have different alkane chain lengths [52]. The study found that the monomers have antibacterial activity against *S. aureus* and *E. coli*, and the antibacterial activity of the monomers is not positively correlate.

Miyazak et al. developed an acrylic acid monomer 2-(tert-butylamino)ethyl methacrylate (TBAEMA) containing a secondary amine motif in the side chain [53]. In this study, the authors believed that TBAEMA is a quaternary ammonium salt. We believe that when the external environment is acidic, the secondary amine motif may carry a positive charge and can exhibit similar functions to QACs (Fig. 4). Experimental results showed that as the content of TBAEMA in the cement increased, the antibacterial activity also increased. In this work, the authors fully discussed the mechanism of antibacterial activity, and they found TBAEMA and amino motifs produced by hydrolysis through mass spectrometry, which may be the cause of antibacterial activity. At the same time, we believe that these low-dose compounds may produce antibacterial activity, but the secondary amine motif on the surface of the cement also contributes to the reduction of bacteria.

Bone cement containing QACs has good antibacterial activity, but the destruction of the cell membrane by QACs lacks selectivity, which may also cause damage to normal cells, manifesting as high hemolytic activity. Therefore, when using it, it is necessary to carefully consider the amount of QACs used or the degree of substitution, achieving a balance between antibacterial activity and safety.

### Antimicrobial peptides

Antimicrobial peptides (AMPs) are a class of peptides with antibacterial activity [54–56]. The number of amino acid residues in antibacterial peptides ranges from 10 to 60, and almost all AMPs contain cationic amino acid residues such as lysine and arginine. Unlike antibiotics that target intercellular functions, AMPs mainly interact with the lipid membrane surface of Gram-negative and -positive bacteria. The cationic groups allow electrostatic interaction with the negatively charged bacterial cell membrane, while the hydrophobic segments facilitate insertion and disruption of the cell membrane, leading to physical damage to the cell. This unique mechanism has been shown to be unlikely to cause antibiotic resistance.

It can be seen that the antibacterial mechanism of AMPs is not only dependent on cations, but also closely related to the spatial conformation of the peptide, which is different from that of cationic polymers. Therefore, some well-studied AMPs have a broad antibacterial spectrum and are less toxic to normal cells. Due to their broad antibacterial spectrum and difficulty in developing resistance, they have become a highly promising new class of antibacterial agents. In recent years, researchers have conducted many studies on the effectiveness of AMPs in bone cement, and the results show that both PMMA bone cement and calcium phosphate bone cement loaded with antibacterial peptides have antibacterial activity.

**Fig. 5:**
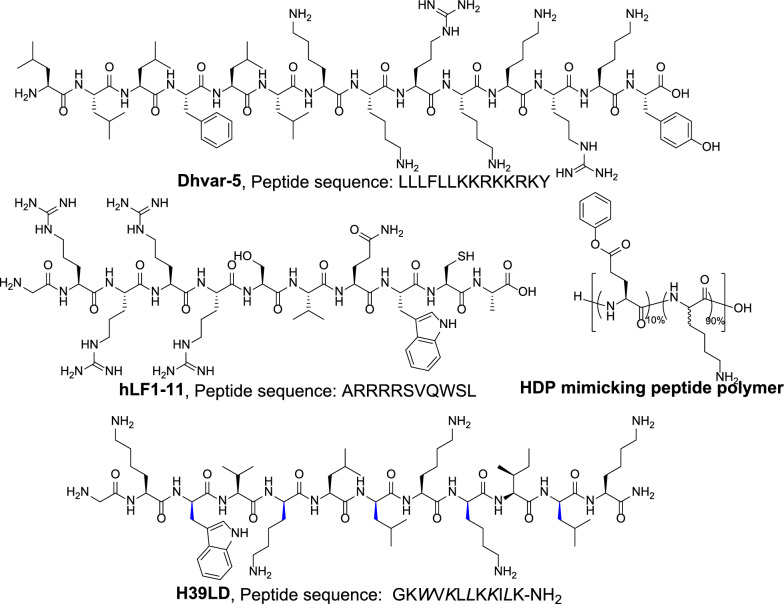
Antimicrobial peptides (D-amino acids are shown in italic)

Wuisman and his team were pioneers in research related to antimicrobial peptide-loaded bone cement [57–62]. In 2003, Wuisman et al. studied the release kinetics of the antimicrobial peptide Dhvar-5 (Fig. 5) loaded into PMMA bone cement. The results showed that the release kinetics of the peptide depended on the amount of peptide added, i.e., the higher the peptide incorporation rate, the higher the proportion of peptide released. Subsequently, they conducted studies demonstrating that Dhvar-5 can increase the porosity of bone cement and enhance the release of gentamicin from gentamicin-loaded bone cement. We believe that this may also be related to drug cluster interconnectivity [3, 63, 64].

In addition, they conducted in vitro release studies and in vivo antibacterial studies on calcium phosphate bone cement loaded with the antibacterial peptide hLF1-11 (Fig. 5). The results showed that this degradable bone cement loaded with antimicrobial peptides is feasible for antibacterial use. Studies have shown that hLF1-11 can not only be released but also effectively reduce the development of osteomyelitis in a rabbit model when loaded into bone cement. AMPs are effective against MRSA, and Wuisman et al. conducted infection treatment studies using hLF1-11-loaded bone cement in a rabbit model of MRSA osteomyelitis. The results showed that the AMPs were effective against infection, with antibacterial activity comparable to gentamicin.

Subsequently, to further investigate the basis of the antibacterial activity of antimicrobial peptide-loaded bone cement, Wuisman et al. also conducted in vivo release studies of hLF1-11-loaded bone cement. The studies showed that there were similar burst release characteristics in vivo as in vitro. We believe that the reason why antimicrobial peptides do not exhibit antibacterial activity comparable to gentamicin may have less to do with the antibacterial activity of the peptides themselves and more to do with their stability in vivo.

While antimicrobial peptides have good application prospects, they also have some inherent shortcomings, such as high production costs and poor stability, which can be easily degraded by proteases in vivo. To address the issue of high production costs of AMPs, Liu et al. designed antibacterial peptide polymer, a method suitable for large-scale and low-cost preparation of antibacterial peptides. Liu et al. studied the mechanical strength and antibacterial activity of HDP mimicking peptide polymer-loaded bone cement (Fig. 5) [65]. They found that the addition of peptide polymer had little effect on mechanical strength, and both in vitro and in vivo studies demonstrated good anti-MRSA activity of the bone cement loaded with antibacterial peptide polymer. The study by Cěrovský et al. considered the stability issue of natural antimicrobial peptides and introduced D-amino acids into the peptides to enhance their stability against proteases [66–68]. Their study found that although the MIC value of the antimicrobial peptides was higher than that of vancomycin and gentamicin in vitro, the calcium phosphate bone cement loaded with antibacterial peptides exhibited higher antibacterial activity in vivo than bone cement loaded with antibiotics. In addition, they also found that PMMA bone cement loaded with antimicrobial peptides could prevent the formation of bacterial biofilms on the surface of the bone cement.

**Fig. 6:**
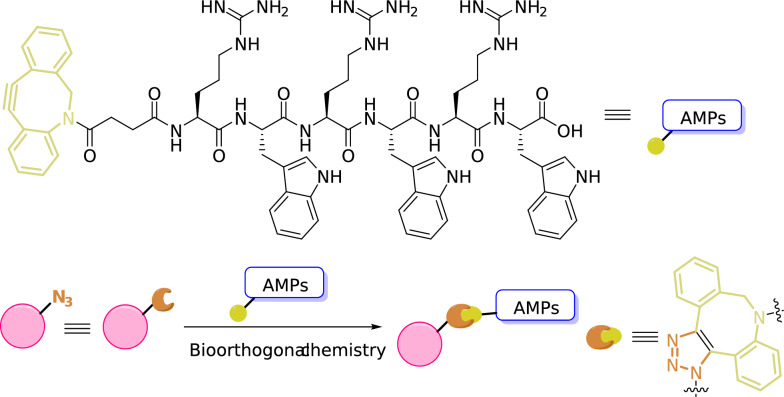
Using bioorthogonal chemistry to immobilize antimicrobial peptides on the surface of PEEK

In addition to introducing D-amino acids to enhance stability, immobilization of antimicrobial peptides is also an effective method to enhance stability or duration of action [69]. Geng et al. covalently conjugated antimicrobial peptides to the surface of bone implant PEEK materials through bioorthogonal chemistry (Fig. 6). Studies have shown that this material conjugated with peptides exhibits antibacterial activity. AMPs contain many reactive functional groups, and although there is currently no bone cement modified through covalent bonding peptides, we believe that the cross-linking of peptides with bone cement based on bioorthogonal chemistry can help promote the development of ABCs.

### pH-switchable antibacterial agent

As mentioned in previous sections, positively charged QACs and AMPs can disrupt cell membranes, but these positively charged antibacterial agents also act on the cell membranes of normal cells, often manifesting as hemolytic activity. To address this issue, researchers have developed pH-switchable antibacterial agents. Bacterial infection creates a locally acidic special pathological environment. Based on this special pH change, researchers have designed antibacterial agents that can respond to pH changes, and pH reduction can enhance the antibacterial activity of the agents. Currently, this novel antibacterial agent has not been studied in bone cement, but we believe that such antibacterial agents are likely to play an important role in the research and application of antibacterial activity of bone cement.

**Fig. 7:**
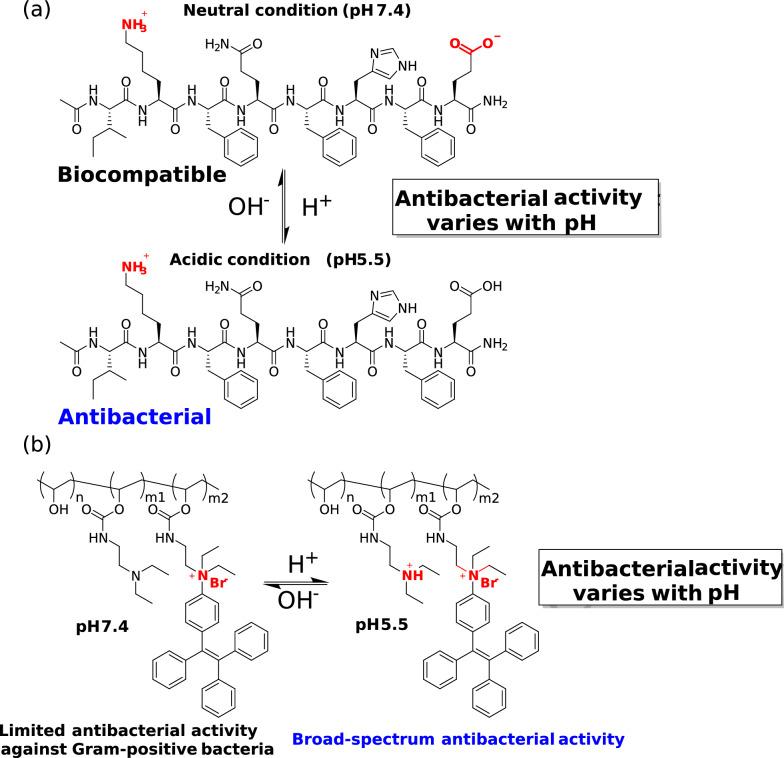
pH-Switchable antibacterial agents of peptide (**a**) and polymer (**b**)

Li et al. designed a pH-responsive adjustable antibacterial octapeptide [70]. We can notice that this peptide has an N-terminal amidation structure and contains lysine and aspartic acid residues in the main chain (Fig. 7a). Under neutral pH conditions (pH 7.4) in physiological conditions, the octapeptide exhibits an overall electrical neutrality (zero net charge) due to the electrostatic neutralization between the amino cations of the lysine residue side chain and the carboxyl anions of the glutamic acid residue side chain, and the peptide has the best biocompatibility.

Under acidic conditions caused by bacterial infection (pH 5.5), the charge of the peptide undergoes a sharp change. First, the carboxylic acid group of the glutamic acid residue side chain is protonated, resulting in the transformation of the electrically neutral octapeptide into a positively charged peptide, exhibiting antibacterial activity similar to that of antibacterial peptides. Based on this novel pH-switchable antibacterial peptide, they prepared hydrogels and used them for wound infection treatment studies. Similar to this research approach, researchers have also developed pH-switchable antibacterial peptides such as antibacterial 5-peptides [71]. As orthopedic implants increasingly emphasize biosafety, it is necessary to incorporate pH-switchable antibacterial peptides into the related research of antibacterial bone cement.

In addition to novel peptide-based pH-switchable antibacterial agents, Ren et al. designed poly(vinyl alcohol) derivatives (PVA–TPE) that can regulate antibacterial activity with pH changes [72]. PVA–TPE exhibits antibacterial activity against Gram-positive bacteria only under neutral pH conditions, but when the pH is reduced to 5.5, the tertiary amine on the polymer side chain is protonated, increasing the overall positive charge, making PVA–TPE exhibit broad-spectrum antibacterial activity against both Gram-positive and negative bacteria (Fig. 7b). Their studies have also shown that PVA-TPE has low cytotoxicity to mammalian cells L929, HepG2, and red blood cells.

We hypothesize that the introduction of both positively charged motifs (such as quaternary ammonium salt motifs) and carefully considered negative charged motifs on the surface of bone cement may reduce the hemolytic activity of quaternary ammonium salts and facilitate the development of novel pH-switchable antibacterial bone cement with good biosafety.

### N-halamine

*N*-Halamines are a novel class of antibacterial agents that can be typically categorized into three types: amine *N*-halamines, amide *N*-halamines, and imide *N*-halamines [73, 74]. The structural stability hierarchy among these three classes is amine *N*-halamines > amide *N*-halamines > imide *N*-halamines. The halogen in *N*-halamines can include chlorine, bromine, or iodine, with chlorine being the most studied and applied. Additionally, halamines can be further classified as cyclic or acyclic based on their parent nuclear structure, with cyclic halamines exhibiting better stability. The antibacterial mechanism of *N*-halamines is believed to involve release killing, contact killing, and transfer killing [73]. We tend to believe that transfer killing can be encompassed within the first two mechanisms, therefore favoring release killing and contact killing as the primary antibacterial mechanisms of *N*-halamines.

Due to their excellent antibacterial properties, materials containing halamines have been widely studied and applied in fields such as textiles and coatings. For example, Goddard et al. utilized Antimicrobial *N*-halamine Modified Polyethylene to fabricate food packaging materials [74], while Sun et al. designed Acyclic *N*-halamine-immobilized polyurethane, which demonstrated antibacterial activity and the prevention of biofilm formation [75]. In another study by Sun et al., they developed polymeric *N*-halamine latex emulsions for the production of antibacterial coatings, which involved the use of cyclic *N*-halamines with good stability that can maintain antibacterial activity for over a year [76]. These studies fully demonstrate the excellent antibacterial applicability and acceptable safety of *N*-halamines.

Halamines also have potential applications in the biomedical field. For instance, Li et al. reported the use of halamine-containing materials for antibacterial purposes in dental implant materials [77]. In another study, researchers functionalized the surface of Ti metal with *N*-halamine, resulting in implants that exhibited good antibacterial properties and osteogenic activity in vitro [78].

**Fig. 8:**
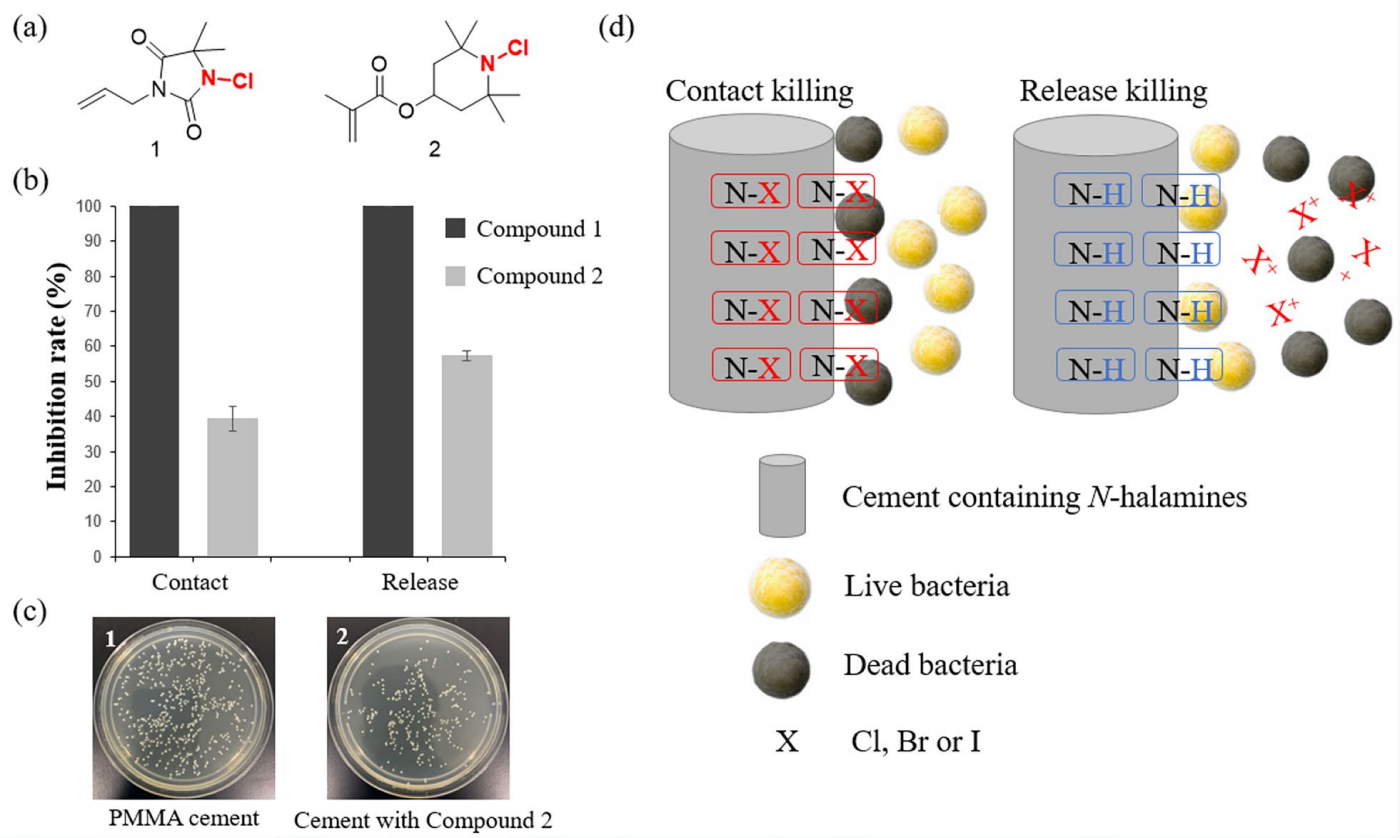
ABCs prepared with new polymerizable *N*-halamine compounds show good antibacterial activity. Chemical structure of *N*-halamine monomers (**a**), inhibitory rate against *S. aureus* (**b**), images of the bacterial colonies formed by *S. aureus* contacted with bone cement with 15 wt% compound 2 (**c**) and the antibacterial mechanisms of* N*-halamine(**d**)

These studies have collectively demonstrated the excellent potential of *N*-halamine-containing materials for use in antibacterial implantable materials. Therefore, we believe that halamines have the potential to serve as antibacterial agents for bone cement. Based on this consideration, our research group has discovered two promising polymerizable monomers containing halamines and used them to prepare halamine-containing antibacterial bone cement. Compound 1 incorporates the amide *N*-halamines, while compound 2 contains relatively stable *N*-halamines (Fig. 8). We aim to investigate whether these two different halamine structures exhibit antibacterial properties in bone cement. We added 15 wt% monomer to the liquid phase in the manner reported in the literature to produce bone cement [79]. Initial research findings suggest that these new cements exhibit excellent contact antibacterial activity and release antibacterial activity against *S. aureus*. As expected, amide *N*-halamines, due to the unstable nature of halogens, exhibited higher antibacterial activity at the same dosage, both in terms of contact and release antibacterial activity. More detailed research on the antibacterial activity, mechanical strength, and biosafety of the *N*-halamine-containing bone cement is the focus of future studies.

### Non-leaching acrylic monomers

**Fig. 9:**
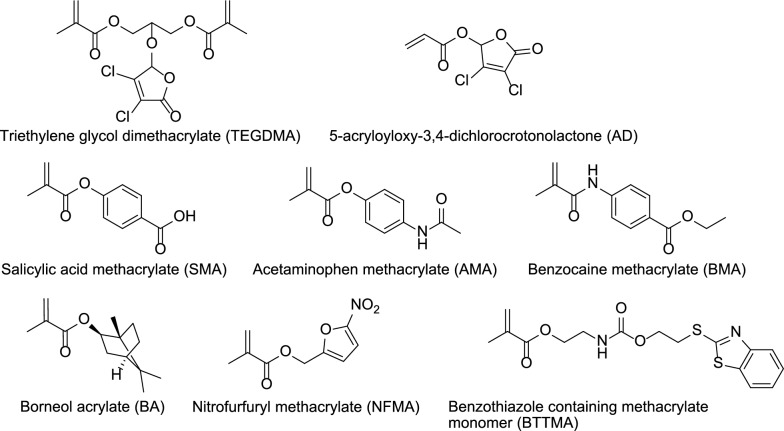
Reported acrylic monomers

The bone cement prepared using non-leaching acrylic monomer is referred to as non-leaching bone cements (NLBCs) [79]. The antibacterial mechanism of NLBCs is believed to be contact killing. For example, QACs antibacterial agents discussed in the Sect. 3 can be classified as non-leaching antibacterial acrylic monomer, and their antibacterial mechanism involves destroying cell membranes. The ABCs with covalently attached antimicrobial peptides that we envisioned in Sect. 4, if they are designed and manufactured, can also be classified as NLBCs. However, many reported non-leaching acrylic monomers are cyclic organic molecules (Fig. 9), and the antibacterial mechanisms of these agents are still unclear [79–87]. Some believe that they may affect the activity of certain enzymes on the cell surface [79]. Surprisingly, research has shown that the addition of non-leaching agents can enhance the mechanical strength of bone cement [85, 86]. We have discussed these in detail in our previously published review articles [79], so we will not elaborate further here.

### Antimicrobial enzymes

Antimicrobial enzymes refer to proteins or enzymes with antibacterial activity, such as lysozyme and Lysostaphin. Research and applications of antimicrobial enzymes in fields such as food and medicine have been ongoing. Therefore, this review will only briefly discuss some recent work instead of providing a comprehensive overview. Researchers have prepared complexes of lysozyme and Poly(lactide-co-glycolide) using hotmelt extrusion, and studied the stability and release properties of the enzyme [88]. The results showed that lysozyme could be completely released while maintaining its activity. Similar studies have involved the preparation of block copolymers containing lysozyme through the solvent extrusion method [89]. In these studies, lysozyme was physically incorporated into the polymer, resulting in non-injectable enzyme-loaded materials. Some studies have designed injectable polymers, in which antimicrobial enzymes are chemically crosslinked to the polymer. Lysostaphin is a metallo-endopeptidase produced by Staphylococcus simulans, and García et al. designed a Lysostaphin and BMP-2 co-delivery gel [90]. This hydrogel, constructed using four-arm maleimide–terminated PEG (PEG-4MAL) macromere, can be used to reduce *S. aureus* infection and regenerate critical-sized segmental bone defects. Recently, Qu et al. used 4-arm-PEG-NHS to construct an injectable lysozyme hydrogel, which was successfully used for corneal stroma defect repair and rapid vision restoration [91].

To date, antimicrobial enzymes have not been incorporated into the research of antibacterial bone cement. We believe that this may be due to the severe preparation conditions (high temperature, hydrophobic environment, etc.) of PMMA bone cement, which hinder the use of antimicrobial enzymes. Inorganic bone cements, exemplified by CPC, do not produce heat during application, making them an ideal candidate for incorporating enzymes. Furthermore, the use of gentle, water-based liquid phase agents, like calcium phosphate, for the development of antimicrobial enzyme-containing bone cement exhibits significant feasibility, as the liquid phase of inorganic bone cement primarily consists of water, allowing enzymes to be incorporated into either the solid phase or dissolved directly within the liquid phase for utilization. In addition, we can also consider integrating antimicrobial enzymes into bone cement through hydrogels to ensure that lysozyme does not denature. In conclusion, antimicrobial enzymes are efficient and biocompatible antibacterial agents that have the potential to play a role in the research of antibacterial bone cement.

### Future perspectives

For decades, antibacterial bone cements have been widely studied, both in academic and medical communities. Since the first report of antibacterial bone cements in 1970 [92], with the continuous development of materials science, pharmacology, etc., various new bone cements have been designed, manufactured and clinical used, with better antibacterial activities, mechanical strength, biocompatibility [93–99]. These studies provide more choices and confidence for humans to overcome orthopedic infections.

Bacterial resistance has become a global challenge. Increasing the dosage of antibiotics can enhance the antibacterial activity of ABCs, but it also leads to mechanical degradation, drug toxicity and even more severe bacterial resistance. Therefore, the design and use of new antibacterial agents are important ways to solve this war against bacterial infections. In this review, we categorize the antibacterial agents currently used or potentially used in cement into seven categories (Table 3). Some ancient antibacterial agents, such as silver salts, have achieved a balance between antibacterial activity and biosafety by combining the latest drug delivery methods suppressing the burst release of silver ions. Antimicrobial peptides are attractive new antibacterial agents, and bone cement containing antimicrobial peptides has been studied for decades. Some inherent disadvantages of antimicrobial peptides, such as easy proteolysis by proteases and hemolytic activity, can be overcome by combining new technologies. For example, by immobilizing the peptide on the surface of the implanted material via bioorthogonal chemistry, its stability is improved; by precise design, pH-switchable antibacterial peptides can be produced to improve the biosafety of antibacterial peptides. The use of antimicrobial peptides and enzymes is also suffering from the high cost of synthesis, therefore, efficient peptide and protein synthesis technology should also continue to be researched and applied [100–104]. These new technologies urgently need to be introduced to the research of antimicrobial peptide-containing bone cement. Other types of new materials also promote the development of ABCs, such as controlled release material, nanomaterial and so on [105–117]. Quaternary ammonium compounds and antimicrobial enzymes are also new antibacterial agents that have attracted increasing attention in recent years. In addition, biodegradable polymers, including chitosan and its derivatives, are also bright new antibacterial agent candidates [47]. The knowledge gained from these studies provides powerful support for the research of new ABCs. These new discoveries have provided a fresh perspective and pushed forward the horizon for addressing orthopedic infections. Back to the beginning, to address the issue of antibiotic abuse, we must embark on developing novel antibacterial agents. In this endeavor, it is imperative that we actively consider a comprehensive safety evaluation framework for these new agents, incorporating regulatory considerations to preemptively prevent potential misuse or abuse of these drugs [118].

**Table 3:** Summary of the novel antibacterial agents-loaded cements in the review

Antibacterial agent type	Advantages	Shortcomings
Metallic antibacterial agents	Broad antibacterial spectrum and low cost	Biological toxicity
Cationic polymers	Broad antibacterial spectrum and low cost	Hemolytic activity
Antimicrobial peptides	Broad antibacterial spectrum, effective against drug-resistant bacteria, good biocompatibility	High cost, easily degradable
pH-switchable antibacterial agents	Good biocompatibility	High cost
N-halamines	Broad antibacterial spectrum and low cost	Biological toxicity
Non-leaching acrylic monomers	Low cost and easy to operate	Complex monomer preparation
Antimicrobial enzymes	Broad antibacterial spectrum and good biocompatibility	High cost, easily degradable

We firmly believe that the investigation of novel antibacterial agents, coupled with their comprehensive evaluation in conjunction with bone cement, holds significant potential in propelling orthopedic science forward and revolutionizing surgical procedures. The development and utilization of bone cement formulations enriched with these innovative antibacterial agents are poised to significantly enhance the management of orthopedic infections.

## Data Availability

No datasets were generated or analysed during the current study.
